# Assessment of knowledge, attitude and practice (KAP) regarding siddha medicines by veterinarians in Tamil Nadu: A cross-sectional observational study

**DOI:** 10.6026/973206300212956

**Published:** 2025-09-30

**Authors:** Varshini Kumar, Selladurai Elansekaran, Venkataraman Krishnamurthy, Gnanaraj Johnson Christian, Murugan Ramamurthy, Venkatachalam Srinivasan, Gayatri R., Ambalavanan Shakthi Paargavi

**Affiliations:** 1 Department of Noi Naadal, National Institute of Siddha, Affiliated to the Tamil Nadu Dr. MGR Medical University, Tambaram Sanatorium, Chennai-47, Tamil Nadu, India; 2 Department of Veterinary Pharmacology Siddha Central Research Institute, Central Council for Research in Siddha, Chennai-106, Tamil Nadu, India

**Keywords:** Siddha, one health, KAP survey, veterinarians, ethnoveterinary, *mattu vagadam*

## Abstract

The one health approach is gaining momentum in India, prompting interest in traditional systems like Siddha and Ethnoveterinary medicine for animal care due to their affordability, safety, and holistic principles. This study explores the application of Siddha medicine and ethnoveterinary practices in veterinary care, drawing insights from ancient texts such as *Maattu Vagadam*. A KAP survey of 150 veterinarians in Tamil Nadu revealed that 64% had used Siddha treatments, with 88.5% expressing satisfaction and 94.8% reporting no adverse reactions. Despite 68.7% lacking formal training in ethnoveterinary methods, over half of the respondents reported ease in applying external therapies and effectiveness during epidemics. Furthermore, 84.7% supported establishing a dedicated Veterinary-Siddha branch, highlighting the potential for integration into modern veterinary practice.

## Background:

Animal husbandry, which refers to livestock rising, plays a crucial role in producing a variety of food products with high nutritional values and offers a variety of other services, including providing draught power, serving as a means of transportation, enriching poor soils with nutrients, generating income and diversifying economic activities, and acting as a form of financial capital [1]. Livestock sustains the livelihoods and food security of nearly a billion people worldwide and accounts for 40% of the value of agricultural output worldwide [2]. India has the largest livestock inventory in the world and generated outputs worth Rs. 11596.36 billion according to the 2019 census and this sector contributes about 5.63% to Tamil Nadu's Gross State Value Added (GSVA) and 43.70% to agriculture and allied activities [3]. Intensive farming is encouraged due to the increasing demand for animal protein in developing countries and these results in antibiotic residues in products produced by animals, which frequently propagate in fresh meat and milk products, ultimately leading to antibiotic resistance [4, 5-6]. It increases their sensitivity to viruses and bacteria and prone to several diseases and conditions that may affect their well-being [7]. Potential health effects from farm emissions are diverse and include zoonotic infections, which are naturally transmitted between animals and humans [8]. Of the 1,407 known human pathogens, 816 are zoonotic, including life-threatening diseases such as plague, Nipah virus, coronavirus, and rabies [9, 10]. The prevalence of various diseases in livestock is a foot-and-mouth disease at 21%, blue tongue at 28%, classical swine fever at 31%, and porcine circovirus at 43% in India [11]. It affects the economy, animal welfare, the environment, and public health, so with that in mind, antibiotic resistance is seen as a "One Health subject" that involves interactions between humans, animals, and the environment as a cause and solution [12]. In the Indian context, the One Health approach is strategically gaining importance among all stakeholders, including public health professionals, veterinarians, healthcare providers, policymakers, and researchers. It emphasizes the importance of collaborative efforts at both regional and national levels to garner more support for integrated disease prevention and to address the consequences of high-impact pathogens of medical and veterinary importance [13, 14]. These issues have ultimately hindered the exploration of alternative herbal-based medicines mainly Siddha which are economical and safe in Tamil Nadu [15, 16-17]. Siddha science is the traditional system of medicine that revolves around various concepts such as 96 *thathuvams* (Basic principles), *Panchabhootham* (5 elements), 7 *Uyir* kattugal (7 Physical constituents), and Mukkuttram (3 humors - Vatham, Pitham & Kabam) [18]. A branch of Siddha medicine focuses on the treatment and prevention of diseases in animals using herbal remedies, natural therapies, and holistic approaches similar to those used in human Siddha medicine which are particularly seen in the ancient treatise called *mattu vagadam* [19]. However, there is a lack of data on the state of Siddha in the veterinary domain. Therefore, it is of interest to document the role of Siddha medicine in veterinary practice and the demographic trends associated with its use.

## Materials and Methods:

## Ethics and data production:

This cross-sectional observational study was approved by the Institutional Research Review Board and Institutional Ethical Committee of the National Institute of Siddha, Chennai, India, IEC NO: NIS/24/IEC/2023/MP/54. It was registered in the Clinical Trial Registry of India No: CTRI/2023/10/058219. Before data collection, participants were informed of their anonymity and provided consent through an accompanying page in the data collection form. Tracking data back to specific persons was, not possible in the data sets. The additional data set gave background information on the study, research contact data, and assurance of anonymity.

## Data collection:

The questionnaire was intended to get data from professional veterinary practitioners in Tamil Nadu. Based on the statistics of 2020 [20], veterinarians of all specializations registered in Tamil Nadu were 6836. Due to data protection reasons, it is not possible to receive a full list of veterinarians from responsible authorities. Therefore, reaching the entire target group could not be an objective of this study. We distributed the questionnaire survey via Google Forms to the participants through e-mail and other social media channels. To be eligible, the participants were required to be employed in Tamil Nadu. Some of them did not respond and a few participants responded twice to the same Google form as it contained pilot responses as well. A total of 174 responses were collected and one of them didn't give his consent. The link prohibited participants from responding again to avoid multiple responses from the same individual. After eliminating duplicate responses and calculating the total responses, we obtained 150 effective responses. This study was conducted from November 15, 2023, to January 9, 2024.

## Sample size calculation:

Among the approximate 7000 professional veterinarians who have registered with Tamil Nadu State Veterinary Council, the number of subjects needed for this study, a sample size calculation was performed assuming a 10% margin of error with a design effect of 1.5 and if 50% among them have a correct knowledge with a confidence level of 95%, based on this calculation and assuming a low response rate, the required sample size is calculated as 143 with a round figure of 150 and convenient sampling technique was used.

## Survey design:

A general literature review was carried out to identify the usage of ethnoveterinary medicines. The questionnaire's structure was well-versed by understandings obtained from qualitative interviews with veterinary educators and animal practitioners from various institutions and pre-testing with that questionnaire was done among 23 veterinarians initially. The questions were sent to the participants individually and were filled up by them. This questionnaire-based online survey contained a total of 28 self-explanatory questions to collect demographic characteristics and assess the KAP in Siddha Medicine. The questionnaire itself was designed to encompass a series of close-ended questions as they are less time-consuming than descriptive questions with an expected completion time of 10 minutes and 3 open-ended inquiries regarding the necessity for data. The first section comprises questions related to demographic information such as name, gender, age, experience, educational qualification, area of expertise, type of employer, and workplace location. The second section focuses on participant's usage of Siddha medicine for their pets. In this dichotomous question, when answered 'yes,' the next practice-related section would be continued. If 'no', it would lead to the next section skipping five more practice-related questions. Likert scales and rating scales were also used in this section. The other sections addressed knowledge and behavioral aspects using multiple-choice, open-ended, and drop-down questions. The multiple-choice options were based on the literature review and were completed by an open field for individual-specific additions.

## Data analysis:

After data collection, all answers were summarised in a table (Microsoft Excel 2013 version 15.0). Afterward, invalid data sets were erased (blanks from those not meeting the inclusion criteria). Data sets with partly blank answers were used for further calculation, altering the statistics. The open questions asking for treatment modalities with the most advantages or disadvantages were categorized using the given treatment modalities of the questionnaire. All open questions were processed using descriptive statistics. Statistical analysis was done using SPSS software. Metric data (item "age and years of experience") were described using mean, minimum, and maximum. All other items were described as categorical data, including "other" or "blank". Answers were described in absolute and relative numbers. Relative numbers were based on the number of "not blank" answers as a statistical population. Demographic data were analyzed to calculate whether the Veterinary practitioners were influenced to use the veterinary Siddha medicine to complement their regular practice (user versus non-user). For metric data, the chi-square test was used.

## Results and observation:

## Demographic information:

The response rate for this survey could not be calculated due to the distribution method described above. Table 1 (see PDF) summarizes the demographic characteristics of the participants. Among 150 participants, 83.3% (n=124) of men were responded. The mean age was 39.4 years with a minimum age of 22 years and maximum age of 75 years. A sizable number of participants (n=89) completed their degrees at Madras Veterinary College (MVC), Chennai except 4 indicated by the 'others' category obtained from universities outside of Tamil Nadu. Among these, two graduated from the Indian Veterinary Research Institute (IVRI), Izatnagar, while the remaining two earned their degrees from the Post Graduate Research Institute in Animal Sciences (PGRIAS), Kattupakkam, and the Kerala Veterinary and Animal Sciences University (KVASU), Kerala. For the area of expertise, participants identified as practitioners' represented 58.7%, academic faculty 23.3%, researchers 8.7%, and 'others' 9.3%. The participants' years of experience averaged 12.4 years, with a maximum of 49 years and a minimum of 0 years, indicating the presence of freshers in the field. Geographical distribution indicates that 22 participants are from the western side of Tamil Nadu, 45 from the central region, 35 from the southern part, 39 from the northern part, and 9 from other parts of Tamil Nadu and the majority belongs to Salem and Chennai district. Figure 1 provides a diagrammatic representation of the usage of Siddha medicine across different districts.

## Employment of Siddha medicines in pets:

Out of all 150 given answers for this item, 64% (n=96) reported employing Siddha medicines for their pets, while 32% (n=54) did not employ Siddha medicines for their pets. The insignificance in the statistical analysis of the demographic data may be due to the small and purposive samples used in the study. The most frequently mentioned indications were digestive disorders (49.3%) specifically diarrhea followed by udder diseases (24.6%) listed in Figure 2 (see PDF). Among 96 Participants who were practicing Siddha, 89% (n=85) participants were satisfied with the results of Siddha medicine, 4 % (n=4) of participants were not satisfied and 7% (n=7) of participants were having mixed feelings. For the Likert scale satisfactory scoring 8.6% (n=8) participants stated 0-3, 35.5% (n=22) participants stated 4-6, and 67.8% (n=63) participants stated 7-10 times satisfied with the outcomes of Siddha medicines. Table 2 (see PDF) outlines the conditions treated with Siddha and ethnoveterinary medications. *Thayir Chundi Chooranam* and a paste made from *Aloe vera* and *Curcuma longa* are the most frequently used Siddha and ethnoveterinary medicines by professional veterinarians for treating pets.

## Information pathways, reasons for employing Siddha medicines, and challenges faced by veterinarians:

Table 3 (see PDF) depicts absolute and relative numbers of information pathways used by veterinarians, advantages and disadvantages faced by them. Professors and seniors played a vital role in the knowledge-gaining process. Cyberspace, relatives, medical representatives, Siddha doctors, Government, and private pharmaceuticals are stated in the "others" column. The most frequently cited reason for using Siddha medicines was "low cost" (66%) which is followed by no side effects (62.7%). In the "others" category, participants mentioned its ability to combat antimicrobial resistance and its additive effects. Additional benefits mentioned were its role as an immunomodulator, the supply of Siddha medicines by TNMSC, clinical effectiveness, acceptance of plant-based extractions by farmers, use as supportive therapy in combination with antibiotics, minimal impact on production and reproduction, tolerance by livestock, acceleration of recovery and healing, alignment with traditional medicine beliefs, and the availability of readily accessible drugs in the kitchen for emergency use. Slow recovery (54%) and lack of knowledge (54%) about drugs are the most cited disadvantages by them. In the "others" category, the most frequently cited disadvantages included a lack of scientific research and evidence, the need to administer the medicines orally, the absence of Siddha in university curricula as a dedicated course module, lack of specificity in treatment, difficulty in convincing animal owners of its efficacy, and the unreliability of pharmaceutical companies producing these medicines. Challenges while face the external therapies, "none" being the most mentioned (55.2%) answer. The need for repeated applications and loss of follow-ups were mentioned in the "others" column.

## Siddha in epidemics and adverse drug reactions:

When asked about the role of Siddha medicine during epidemic seasons among the 96 individuals who practiced Siddha, 52% stated that Siddha can be used alongside vaccines and allopathic drugs, 24% said it is very useful, 16% said it is somewhat useful, and the remaining 8% said it was not useful at all. 95% of participants experienced No Adverse Drug Reactions and 5% of participants experienced Adverse Drug Reaction such as intense itching, self-mutilation, diarrhea, skin burning, and ulcers which leads to bleeding but failed to share the drug details.

## Ethnoveterinary training and knowledge of Siddha literature:

Among 150 participants, 31% of participants (n=47) had undergone formal ethnoveterinary training in which 55% of participants (n=26) found it 'very useful', 43% (n=20) of participants were found it 'somewhat useful', 2% of participants (n=1) was found it not at all useful and 69% (n=103) of participants have never experienced such training. 19% of participants were aware of Siddha classic literature *Maattu Vagadam*, 4% of participants were conversant with *Sarabendhira vaidhiya muraikal*, 31.3% of participants were aware of recent day Ethnoveterinary book *Mooligai vazhi kaalnadai maruthuvam*, 61.3% participants knew nothing from the list and remaining 0.7% of participants were aware of other literatures such as *Gunapadam- Mooligai vagupu (Herbals)*, *Gunapadam - Thathu seevam vagupu (Minerals)*, most of the Siddha books from Tamarai publications, Ethnoveterinary medicines for different ailments by National Dairy Development Board, Gujarat.

## Utilization of other AYUSH streams in treating animal diseases:

The participants stated that the majority (52%) did not use any indigenous medicines for their pets. Among those who did, 22% of veterinarians used Ayurveda, 0.7% used Unani, 2% used Yoga and Naturopathy, and 33.3% used Homeopathy. This suggests that these alternative treatments may be effective for managing animal diseases that are challenging to address with conventional allopathic medicine.

## Expectations from Siddha and SWOT analysis:

The majority of participants (58.7%) expressed a preference for combining Siddha medicine with allopathic treatments in the future to achieve better results, 16% of participants expected a complete cure, 11.3% attempted Siddha treatment because allopathy had not been effective in particular cases and 10.7% were interested in exploring safer drug options. Expectations for specific results for particular diseases, along with details about the availability of Siddha medicine, including usage instructions, dosage, and ingredient information, are noted in the "others" column. Most participants (48%) identified the affordability of Siddha as its primary strength. Regarding the perceived weaknesses of Siddha, 48% considered it as native knowledge and for the opportunities found in Siddha, most participants (53%) identified it as a newer scope for research. In assessing threats to the Siddha system, 49% of participants identified the budding of quackery as the primary threat. Table 4 (see PDF) shows the SWOT analysis of Siddha [21].

## Opinions on Siddha veterinary integrated branch in the future:

The majority of 84.7% of participants (n=127) believed the Siddha veterinary integrated branch would be useful in the future. Figure 3 (see PDF) shows the usefulness of the Siddha veterinary integrated branch. Given that Siddha medicine can serve as a preventive measure, reduce treatment costs for poor and marginalized farmers, offer an alternative to antimicrobial resistance, ultimately improve the rural economy, and address the need to enhance the availability of these formulations/, the establishment of an integrated branch is essential. Some suggested that Siddha medicine could be incorporated as a course in the Bachelor of Veterinary Science (B.V.Sc) program, with an emphasis on integrating traditional knowledge into the educational curriculum for improved outcomes. Others propose that Siddha could be developed into a separate degree, similar to those for human medicine. Additionally, some believe it could be integrated with allopathy and homeopathy, while others believe that instead of creating a new branch, integrating Siddha and additional ethnoveterinary knowledge into the mainstream pharmacology and microbiology curriculum within veterinary science could expand their use in veterinary practice. Others view this integration as optional and emphasize the importance of understanding the pharmacodynamics and pharmacokinetics of Siddha medicine. They also stress the need for extensive clinical trials and scientific validation to support an evidence-based approach in incorporating these traditional practices into modern veterinary medicine. Some, however, believe that establishing an integrated branch is not necessary. They argue that it could lead to an increase in quackery, may not be suitable for emergency medicine due to the preference of farmers for fast recovery, and that the lack of transparency in drug components makes it difficult to predict adverse drug reactions (ADR). Table 5 (see PDF), 6 (see PDF) & 7 (see PDF) gave the details of Statistical analysis of Siddha medicine usage and drugs used by Veterinarians.

## Discussion:

A total of 57 distinct cases were recorded as being treated with ethnoveterinary medicines. Among these, a total of 54 varieties of medicines were documented in which 21 types of Siddha formulated medicines and 33 types of other ethnoveterinary and conventional medications were documented. According to the cross-sectional survey conducted across India by Vijay *et al.* the male-to-female ratio and the level of education of the respondents closely align with the current survey. However, there is a difference in the years of experience. Additionally, the major disease condition requiring antibiotic use in bovines in India is mastitis, which is not only the second most common disease but also the leading condition for which antibiotics are used and treated with Siddha medicine by veterinarians. This highlights the growing scope for Siddha medicine in the near future. The information sources referred to by veterinarians on antibiotic use and resistance differ significantly when compared to those related to Siddha medicine [22]. The incidence of diarrhea in cattle calves is found to be 52.51 % in India [23] and the highest calf mortality is due to diarrhea [24]. A recent study revealed that *Thayir Chundi Chooranam*, a herbo-mineral formulation in Siddha, as a novel oral rehydration solution that addresses all aspects of comprehensive treatment for managing dehydration and treating diarrhea [25]. It indicated chronic diarrhea, fever, vomiting, and diarrhea due to indigestion, and sprue, and its physicochemical and phytochemical analysis for the drug validated the traditional claim [26]. In *Inji Chooranam*, *Zingiber officinale* Roscoe is the predominant ingredient and found effective when administered orally to livestock for promoting stomach digestion, treating digestive disorders, relieving flatulence, and acting as an appetizer in cattle [27] Al-Yahya *et al.* and Chits *et al.* clarified that this efficacy is likely due to the antiulcer and gastroprotective properties of ginger [28, 29]. According to Jain *et al.* *Nilavembu Kudineer* is a polyherbal decoction with *Andrographis paniculata* (Burm.fil.) Nees as the chief ingredient concoction are used in the treatment of fever, inflammation, and general debility conditions, formulated to manage Chikungunya and Dengue [30]. So it shows possible action against viral diseases with fever like PPR in goats. Xiong *et al.* clarified that the decoction is prepared from the roots and administered orally to livestock for the treatment of inflammation [31]. *Rubia cordifolia L*. (Rubiaceae) is known for its anti-inflammatory activity [32]. Periyanayagam *et al.* investigated the topical anti-inflammatory activity of Pinda Thailam, which contains aqueous extracts of *Rubia cordifolia L*. (Rubiaceae) and *Hemidesmus indicus* (L.) R. Br (Asclepiadaceae). The evaluation was conducted using the carrageenan-induced paw edema technique in albino rats [33]. The herbal gel formulation demonstrated significant anti-inflammatory effects, comparable to the reference standard Diclofenac sodium gel. Kumar *et al.* explained this leaf juice is applied externally to the affected parts of the foot of animals [34]. Swamy *et al.* explained the indications for *Mathan Thailam* which is a herbo-mineral Siddha formulation used to treat various conditions, including eczema, weeping eczema, itching, wounds, chronic ulcers, folliculitis, and more [35]. The presence of copper from copper sulfate, lauric and capric acids from coconut oil, and various phytochemicals from the leaves of *Datura metel L*. contributes to wound healing and counteracts bacterial and fungal infections. This composition suggests its potential anti-dermatitis and wound-healing properties in animals [36]. Oda *et al.* studies suggested the utilization of Datura in animal health care [37]. Kaushik *et al.* have demonstrated the potential ability of *Tribulus terrestris L*., which is the main ingredient in *Nerunjil kudineer* to inhibit the formation of uroliths in vivo, suggesting its possible effectiveness in managing uroliths in dogs [38]. Verma *et al.* highlight the effectiveness of its leaf juice in treating colic and chronic cough in livestock healthcare [39]. Orhan *et al.* investigated the biological effects of *Centella asiatica (L.)* Urb. These are attributed to its triterpene derivatives like Asiatic acid, madecassic acid, asiaticoside, madecassoside, and brahmic acid and they have neuroprotective properties resulting from positively influencing oxidative stress parameters [40]. Islam *et al.* explained the neuroprotective activity of *Centella asiatica* in cattle [6]. The trial conducted by Priya *et al.* highlights that *Seenthil Chooranam* exhibited a protective effect in broiler chickens against Newcastle disease and reduced mortality rates [15]. In the Siddha system, epidemics and pandemics are referred to as *Uzhi Noi*, *Kothari Noi*, or *Ottu Noi* [41, 18]. Manikkavasagam *et al.* and Prakash *et al.* studies showed that the Siddha system of medicine has accumulated extensive experience in managing endemic and epidemic diseases [42, 43]. Additionally, livestock farmers have developed strategies to control and effectively treat diseases by utilizing their existing local knowledge of plant-based medicines which is more effective in such cases [44, 45]. For instance, in this study, the widely used ethnoveterinary mixture of *Aloe vera*, turmeric, and limestone is more effective in treating mastitis [46]. According to Misra *et al.* Himax is a broad-spectrum ointment for animals that contains phyto actives promoting wound healing [47]. The trial conducted by Huseini *et al.* demonstrated the hepatoprotective efficacy of Liv-52 syrup against cirrhotic liver disease. This suggests that the effects of Liv-52 on liver diseases in animals may be attributed to its diuretic, anti-inflammatory, antioxidant, and immune-modulating properties [48]. Veterinarians based in Salem widely use Siddha medication, likely due to the presence of the Shervaroy Hills and the area's unique environment provides a rich source of traditional medicinal plants that are integral to Siddha practices [49, 50-51]. Many government sector veterinarians found difficulty in using Siddha medicines which are available in their dispensary. As for the literature knowledge, most of them are aware of recently published ethnoveterinary books, which feature the necessity of modernized applicability of drugs mentioned in classical literature. In many cases, medications indicated for treating human diseases are also used to treat similar diseases in animals. However, it is important to note that certain diseases differ between species, like in genetic pathways, but studying similar diseases in both humans and animals can still provide valuable insights into the challenges of those diseases. Understanding these differences, along with factors like species and body size, through integrated approaches can benefit both human and animal health [52, 53]. Given the above concept, the Tamil Nadu Veterinary and Animal Sciences University, has established Ethno Veterinary Herbal Research Centre for Poultry (EVHRCP) in Namakkal on 25.02.2011, the first of its kind for poultry in India with the aim of documentation of ethnoveterinary practices in poultry, validation of ethnoveterinary practices by different in vitro and in vivo methods, and propagation of viable practices to the poultry husbandry [54]. This study is the first of its kind among Ayush doctors and veterinarians and therefore the significance is understood in the research field. Estimated observations and results could not be generalized to the population.

## Conclusion:

The widespread use of Siddha and other ethnoveterinary medicine in Tamil Nadu among veterinarians is shown. Nevertheless, uncertainties in evidence, safety, and qualification for use exist. The present study, with its restrictions, supports the need for a discussion about evidence, official regulations, and the need for official qualifications and integrated branches due to the widespread application of veterinary Siddha medicine. More data about the modes of action and effectiveness of modalities are needed to enable veterinarians to practice evidence-based management to benefit society at large.

## Limitations of the study:

The participants were selected through purposive sampling, which may constrain the results' generalizability. It is acknowledged that an in-depth exploration of this domain may not offer a comprehensive overview of the country's Siddha usage situation. However, the outcomes will offer insights into the gaps and scenarios prevalent at the base level.

## Declarations of interest:

None

## Funding source:

This research did not receive any specific grant from funding agencies in the public, commercial, or not-for-profit sectors.

## References

[1] Tourrand JF (2015). Livestock Farming & Local Development..

[2] http://www.fao.org.

[3] https://www.tn.gov.in/.

[4] Manyi-Loh C (2018). Molecules..

[5] Chowdhury S (2015). Veterinary world..

[6] https://www.jphdc.org/index.php/jphdc/article/view/84.

[7] Scialabba N (2022). Sustainable production and consumption: Low input livestock landscapes..

[8] Wallace RB (2008). Wallace/Maxcy-Rosenau-Last Public Health & Preventive Medicine..

[9] Woolhouse MEJ (2005). Emerging Infect Dis..

[10] Kumar S (2020). Indian J Community Med..

[11] Barman N (2020). Vet World..

[12] Velazquez-Meza ME (2022). Vet World..

[13] Aggarwal D (2020). Indian J Community Med..

[14] Mackenzie JS (2019). Trop Med Infect Dis..

[15] Priya K (2020). J Entomol Zool Stud..

[16] Joshi SS (2015). Indian J Anim Sci..

[17] Karthick M (2024). Asian J Res Zool..

[18] Shanmugavelu M (2003). Directorate of Indian Medicine and Homeopathy..

[19] Nellaiyappa P (2014). International Journal of Pharmacy and Pharmaceutical Research.

[20] https://www.tnsvc.org/register_practitioner.php.

[21] https://www.academia.edu/734795/SWOT_analysis_of_Siddha_medicine.

[22] Deepthi V (2021). Antibiotics (Basel)..

[23] Malik S (2012). Am J Anim Vet Adv..

[24] Mandal A (2019). Indian J Anim Health..

[25] Selvi VGS (2014). Afr J Biol Sci..

[26] Keerthana R (2023). International Journal of Pharmacy and Pharmaceutical Research.

[27] Bhat NA (2023). Heliyon..

[28] Al Yahya MA (1989). Am J Chin Med ..

[29] Chits JK (2020). Molecules..

[30] Jain J (2019). J Ayurveda Integr Med..

[31] Xiong Y (2020). J Ethnobiol Ethnomed..

[32] Wen M (2022). Front Pharmacol..

[33] Periyanayagam K (2004). Ancient Sci Life..

[34] Kumar GMP (2017). Int J Pharm Pharm Sci..

[35] https://books.google.co.in/books?id=qEJ1mgEACAAJ.

[36] Arunadevi R (2020). J Ayurveda Integr Med..

[37] Oda BK (2024). J Ethnobiol Ethnomed..

[38] Kaushik J (2019). Sci Rep..

[39] Verma RK (2014). Asian Pac J Trop Biomed..

[40] Orhan IE (2012). Evidence-based complementary and alternative medicine..

[41] Sambasivam Pillai TV (1947). Dictionary of Medicine, Chemistry, Botany and Allied Science..

[42] https://internationaljournal.org.in/journal/index.php/ijayush/article/view/449.

[43] Prakash P (2021). Biocatalysis and Agricultural Biotechnology..

[44] Ranjan (2016). Indian Journal of Animal Sciences..

[45] Gakuya DW (2011). African journal of traditional, complementary and alternative medicines..

[46] https://agriculture.vikaspedia.in/.

[47] Misra SK (1998). Indian Journal of Indigenous Medicines..

[48] Huseini HF (2005). Phytomedicine..

[49] Alagesaboopathi C (2015). Medicine, Environmental Science..

[50] Selvaraju A (2011). International Journal of Phytomedicines and Related Industries..

[51] Usha S (2015). Journal of Traditional and Complementary Medicine..

[52] Nunney L (2015). Philosophical Transactions of the Royal Society B..

[53] King TA (2021). Emerging Topics in Life Sciences..

[54] https://www.tanuvas.ac.in/nkl_evmpoultry.php.

